# A Reflective Framework for Performance Management (REFORM) of Real-Time Hybrid Simulation

**DOI:** 10.3389/fbuil.2020.568742

**Published:** 2020-09-25

**Authors:** Amin Maghareh, Yuguang Fu, Herta Montoya, Johnny Condori, Zixin Wang, Shirley J. Dyke, Arturo Montoya

**Affiliations:** 1 School of Mechanical Engineering, Purdue University, West Lafayette, IN, United States,; 2 Lyles School of Civil Engineering, Purdue University, West Lafayette, IN, United States,; 3 Department of Civil and Environmental Engineering, The University of Texas at San Antonio, San Antonio, TX, United States

**Keywords:** real-time hybrid simulation, run-time sensitivity indicator, self-tuning robust control, run-time stability threshold, RTHS, REFORM, real-time control, RTHS framework

## Abstract

Currently, the lack of (1) a sufficiently integrated, adaptive, and reflective framework to ensure the safety, integrity, and coordinated evolution of a real-time hybrid simulation (RTHS) as it runs, and (2) the ability to articulate and gauge suitable measures of the performance and integrity of an experiment, both as it runs and *post-hoc*, have prevented researchers from tackling a wide range of complex research problems of vital national interest. To address these limitations of the current state-of-the-art, we propose a framework named Reflective Framework for Performance Management (REFORM) of real-time hybrid simulation. REFORM will support the execution of more complex RTHS experiments than can be conducted today, and will allow them to be configured rapidly, performed safely, and analyzed thoroughly. This study provides a description of the building blocks associated with the first phase of this development (REFORM-I). REFORM-I is verified and demonstrated through application to an expanded version of the benchmark control problem for real-time hybrid simulation.

## INTRODUCTION

Engineers in the coming decades will need to push the boundaries in infrastructure design. New materials, additive construction methods, smart materials and dampers, bio-inspired designs, etc. are being developed to support this vision for an incredible infrastructure of tomorrow. However, realizing the implementation of these novel materials and structure first requires that we provide evidence that they can perform at levels that go well beyond present-day expectations. They must be able to withstand extreme loads under uncertain conditions; they must be proven to sustain the severe loads that earthquakes, hurricanes, tsunamis, and tornadoes frequently impose on our structures (Gardoni and LaFave, 2016). The experiments of today enable the infrastructure of tomorrow. As these transformational concepts are developed for infrastructure systems, the research community demands new testing platforms for dynamic experimentation that are realistic and cost-effective. Experimentation with and validation of such systems within the complex scenarios in which they will operate require a new generation of experimental platforms that are flexible, adaptive, predictive, and safe. As the range of scenarios involving dynamic experimentation necessarily becomes more complex, it is clear that the experimental platforms required to conduct sufficiently deep investigations of these engineering systems do not yet exist (Condori et al., 2020).

Real-time hybrid simulation (RTHS, hereafter) is a powerful cyber-physical technique for dynamic experimentation that allows researchers to study the complex dynamical behaviors of infrastructure systems in realistic scenarios (Karavasilis et al., 2011; Gao et al., 2013). RTHS couples physical specimens and computational models in a single experiment to simulate a complete structural system. Transfer systems (e.g., hydraulic actuators and shake tables) are used to enforce interface conditions, numerical models are used to simulate the virtual components, and measurements are collected to feed into to the numerical model. Control schemes are needed in this closed-loop system to compensate for transfer system dynamics and interactions with physical specimens, ensuring that the entire simulation is conducted safely and in the most realistic manner possible. The entire simulation is executed in real-time (i.e., the test duration is the duration of the extreme event) requiring strict timing guarantees.

Due to deep uncertainties in the physical substructure and transfer system, and the need for aggressive control to maintain stability and safety, advanced non-linear control, uncertainty quantification, estimation and prediction must be employed. Several of these issues have been examined in isolation and thus, RTHS has been a subject for continuous development over the past two decades. For instance, researchers have improved the accuracy and stability of interface condition enforcement, see (Gao et al., 2013; Ou et al., 2015; Palacio-Betancur and Gutierrez Soto, 2019; Tao and Mercan, 2019; Wang et al., 2019; Xu D. et al., 2019). Further, Chae et al. (2013), Chen et al. (2015), Maghareh et al. (2020), and Condori et al. (2020) developed adaptive actuator compensation schemes to achieve improved control of servo-hydraulic systems with non-linearities. Fermandois and Spencer (2017) developed a multi-input, multi-output control design approach with accurate reference tracking and noise rejection. Wallace et al. (2005), Mercan and Ricles (2008), Maghareh et al. (2014), Maghareh et al. (2017), Gao and You (2019), and Xu W. et al. (2019) have also established and validated stability and accuracy metrics to enhance credibility and to encourage much broader applications of RTHS than have been possible to date, for example real-time aerodynamics hybrid simulation by Wu and Song (2019) and Su and Song (2019), experimental testing of spacecraft parachute deployment using RTHS by Harris and Christenson (2019). Nonetheless, despite the potential for using RTHS to conduct low-cost, high-efficiency experiments, the lack of a modular framework that can systematically integrate these existing capabilities, and do so while establishing clear requirements for the safety, integrity, and coordinated evolution of an RTHS experiment, has prevented researchers from tackling a broader range of complex problems.

This study presents the first phase of development and numerical validation of *Reflective Framework for Performance Management* (hereinafter referred to as REFORM) of real-time hybrid simulation. The main objective of this phase of development (REFORM-I) is to define the building blocks and develop a modular architecture that will enable conducting black-box and reference-free experiments safely and with high confidence. A key aspect of this is having the ability to appropriately allocate dedicated resources to control and prediction tasks. In future phases of REFORM development, we will develop and share a highly modular framework capable of (1) exploiting existing prediction, control and model-updating techniques developed by different researchers in the RTHS research community, (2) adapting computational and control workloads and simulation rates, and (3) enabling more challenging and realistic experiments safely and with high confidence. We will make this available through an NSF-funded Research Coordination Network on this topic, known as the Multi-hazard Engineering Collaboratory on Hybrid Simulation (MECHS, https://mechs.designsafe-ci.org/).

## FRAMEWORK DESCRIPTION

**FIGURE 1 |:**
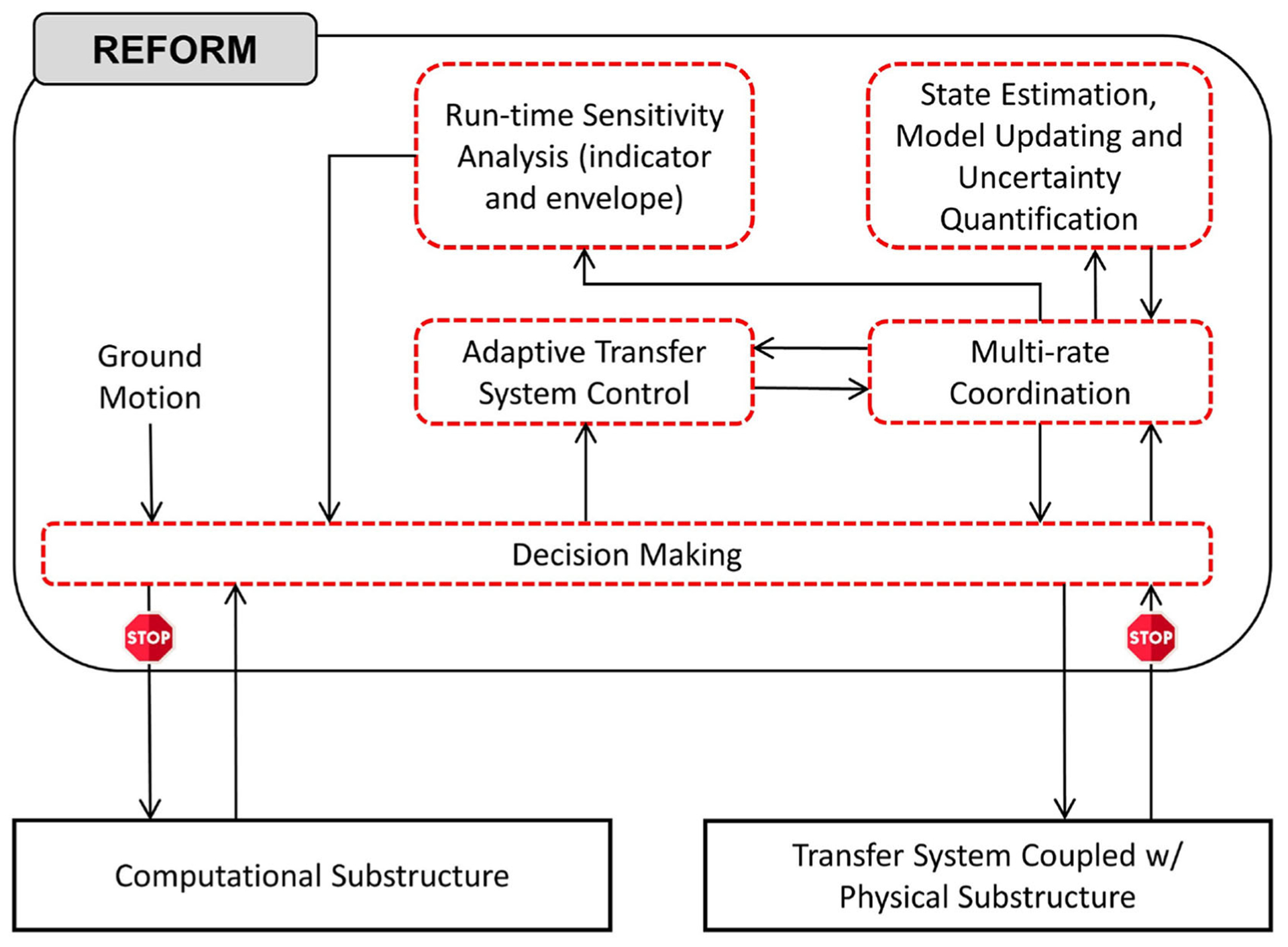
Overview of the first phase of the Reflective Framework for Performance Management (REFORM-I) of real-time hybrid simulations.

RTHS is intended to minimize the need for full-scale dynamic testing (e.g., shake table testing), and as such, these experiments must be performed without a full physical reference experiment. Thus, simulations are characterized by deep uncertainties in the physical substructure, significant coupling/interaction between the physical and computational substructures, and deep uncertainty in the emergent system behavior. When knowledge of the physical specimen, and therefore the reference structure, is limited, such *black-box* and *reference-free* testing necessitates the use of the latest developments in adaptive and robust control systems, prediction and estimation. REFORM-I is, therefore, broken down into five generic building blocks: (1) multi-rate coordination; (2) transfer system control; (3) state estimation and model updating; (4) run-time indicators; and (5) decision making. Figure 1 shows an overview of REFORM-I representation and coordination between these building blocks.

### Multi-Rate Coordination

**FIGURE 2 |:**
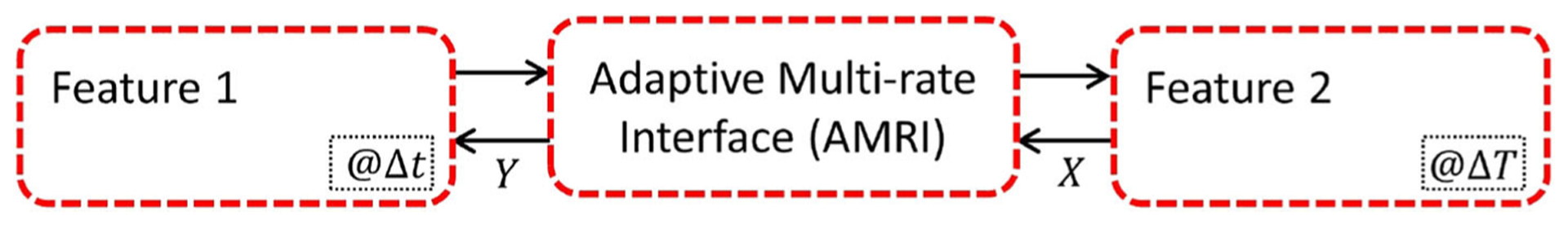
Use of the AMRI for multi-rate coordination in REFORM-I.

In REFORM-I, a multi-rate coordination technique is required to enable users to coordinate the necessary multi-rate functionalities of the various building blocks. This multi-rate coordination block facilitates the use of complex and high-fidelity features. The Adaptive Multi-rate Interface (Maghareh et al., 2016) is utilized to 86 meet this objective. The adaptive multi-rate interface (AMRI, hereafter) was initially developed as a mechanism to facilitate greater fidelity in the computational substructure by running the numerical model at a larger time-interval than what is used for the control system, thereby providing supporting use of a computationally demanding model. In REFORM-I, however, AMRI serves more generally as the multi-rate coordination technique that enable effective communications between feature blocks with different sampling rates, see Figure 2. In this method, after selecting a set of orthonormal bases (e.g., polynomial or exponential) sampling frequency ratio between the feature blocks. Then, a synchronized signal is generated by AMRI at the rate of Δ*t* where *X* is the input signal at coarse time interval Δ*T*, *Y* is the output signal at sub-interval Δ*t*, SFR is the sampling frequency ratio (Δ*T*/Δ*t*), *k* is the number of orthogonal bases used for interpolation, *r* is the number of points used for interpolation, *p* is the compensation coefficient, and *p*Δ*T* is the time to be compensated.

Chebyshev polynomials of the first kind are used as the set of orthonormal bases for interpolation and rate transitioning from Δ*T* to Δ*t*. The polynomials are defined by the following recurrence relation.

(1) $$\{\begin{array}{l} {\text{T}}_{1}(\text{s})=1\\ {\text{T}}_{2}(\text{s})=\text{s}\\ {\text{T}}_{i+1}(\text{s})=2_{\text{s}}{\text{T}}_{\text{i}}(\text{s})-{\text{T}}_{\text{i}-1}(\text{s}) \end{array}$$

**FIGURE 3 |:**
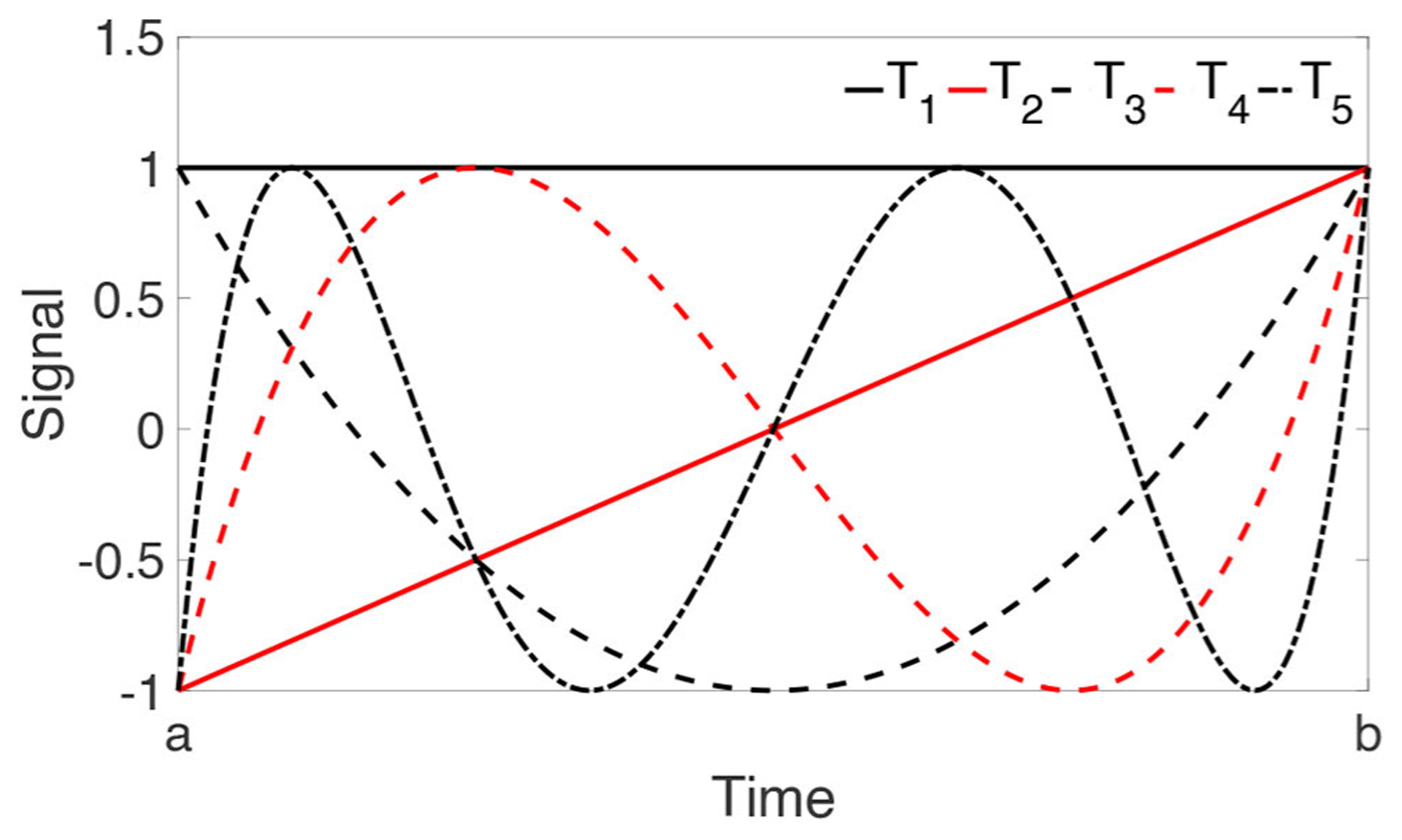
First five adjusted Chebyshev polynomials for a general range of [a, b].

These polynomials are adjusted to be within a general range of [*a*, *b*], where, *a* = (*p* + *r* − 1)Δ*T* and *b* = *p*Δ*T*. For this adjustment, $s=\frac{2x-(a+b)}{b-a}100$ where *x* corresponds to a dummy variable in the range of [−1, 1], see Figure 3. Next, the following linear equation is solved to obtain {β_1_, β_2_ … β_*K*_}.

(2) $${\left(\begin{array}{ccc} (T_{1}[(p+1-r)\Delta T] & \ldots & T_{k}[(p+1-r)\Delta T]\\ \vdots & \ddots & \vdots \\ T_{1}[(p)\Delta T] & \ldots & T_{k}[(p)\Delta T]) \end{array}\right)}_{r\times k}\times \left(\begin{array}{c} \beta_{1}\\ \beta_{2}\\ \ldots \\ \beta_{k} \end{array}\right)=\left(\begin{array}{c} X_{n-r+p+1}\\ \cdots \\ X_{n}\\ \cdots \\ X_{n+p} \end{array}\right)$$

Using the *β* coefficients, the output signal at the coarse time interval Δ*t* can be reconstructed as follows:

(3) $$\text{Y}(\text{h})=\beta_{1}T_{1}(\text{h})+\beta_{2}T_{2}(\text{h})+\cdots +\beta_{k}T_{k}(\text{h})$$

Where

h∈{(n+p−1)ΔT,(n+p−1)ΔT+Δt,(n+p−1)ΔT+2Δt⋯(n+p)ΔT}.

### Adaptive Transfer System Control

This building block is broken down into two main tasks: (1) developing a physics-based, control-oriented non-linear dynamical model of a multi-actuator transfer system coupled with a non-linear physical specimen; and (2) designing a high-precision self-tuning robust controller to accommodate extensive variations in a non-linear control plant, such as non-stationary behavior or component failure.

In RTHS, controllability is a significant property of the transfer system. The key to modeling the control plant is making realistic assumptions within the operating range of an experiment, maintaining the essential dynamics, and discarding the insignificant ones. A fundamental step before developing a control strategy and designing a control law is realizing existing constraints and making realistic assumptions, for instance, the dynamic interactions between physical substructure and transfer system (a.k.a., control-structure interaction, Dyke et al., 1995) and extensive performance variations and uncertainties associated with the control plant (Maghareh et al., 2018a,b; Condori et al., 2020).

In REFORM-I, we have developed deterministic and stochastic physics-based non-linear dynamical models of a servo-hydraulic transfer system coupled with a non-linear physical specimen. These models have been developed for single- and multi-actuator systems and experimentally validated for a single actuator system coupled with a non-linear physical specimen (Maghareh et al., 2018a,b). Transforming the plant model into a controllable canonical dynamical model makes it appealing for developing more advanced non-linear control systems. Adopting these models becomes especially important in two cases: (1) when control-structure interaction dominates the dynamics of the coupled systems; and (2) when the hydraulic system is coupled with a physical specimen with high uncertainty.

**FIGURE 4 |:**
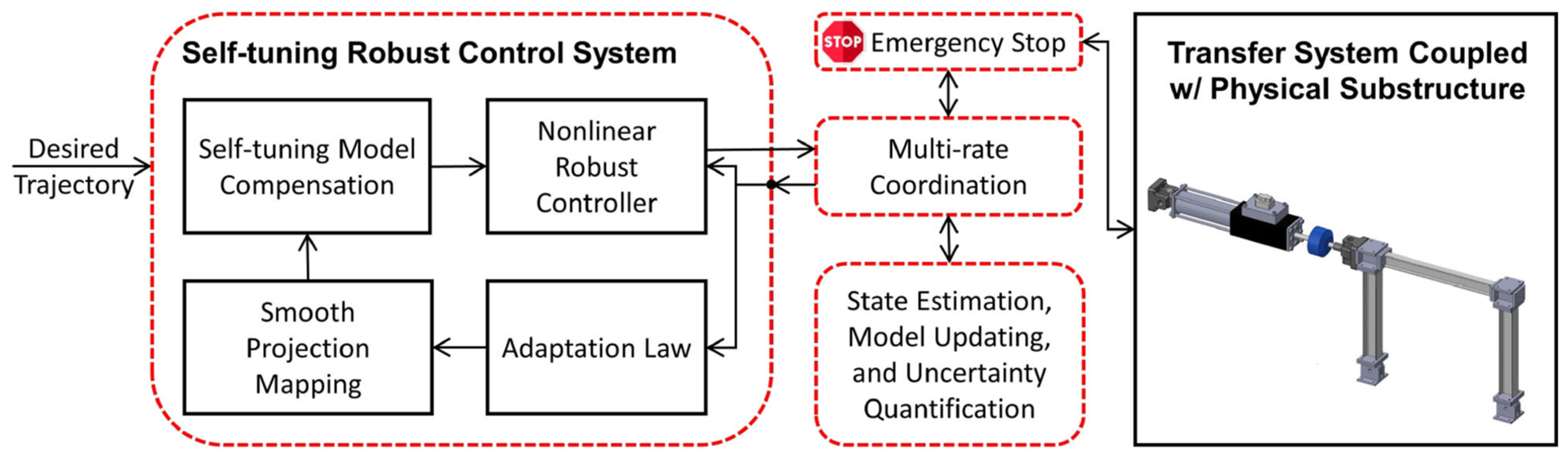
Block diagram of the Self-tuning Robust Control System in REFORM-I.

The physics-based, non-linear dynamical models of a servo-hydraulic transfer system coupled with a non-linear physical specimen serve as the basis for developing an effective control strategy which accommodate highly uncertain dynamics of the control plant and *control-structure interaction* (Maghareh et al., 2018a,b Montoya et al., under review). To illustrate the capabilities required in this building block, we use a model-based, multi-layer nonlinear control system (*Self-tuning Robust Multi-directional Control System*, hereinafter referred to as SCRSys) developed by Maghareh et al. (2020) as the adaptive transfer system control strategy, see Figure 4. This control strategy has been developed, validated and incorporated in REFORM-I to accommodate extensive performance variations and uncertainties in the physical substructure. Our initial experiments with the Self-tuning Robust Multi-directional Control System also suggest that it has significant promise for complex multi-directional scenarios. SRCSys consists of two layers, robustness and adaptation. The robustness layer synthesizes a non-linear control law such that the closed-loop dynamics perform as intended under a broad range of parametric and non-parametric uncertainties. Then the adaptation layer reduces those uncertainties at run-time through slow and controlled learning of the control plant. SRCSys is developed for single- and multi-actuator transfer systems and experimentally validated for single-actuator RTHS experiments (Maghareh et al., 2020).

Here, the layer of adaption is designed to reduce estimation error, while the stability of the layer of robustness remains intact. In an RTHS experiment, there are parametric and non-parametric uncertainties associated with the control plant. Under certain conditions, the layer of robustness filters out the high frequency unmodeled dynamics in the transfer system (Maghareh et al., 2020). However, unless the parametric uncertainties are gradually reduced at run-time by a self-tuning mechanism, they may cause tracking performance degradation. Thus, the layer of adaptation is to suppress the parametric uncertainties, or the parametric variations—e.g., yielding or internal resonance in the physical substructure—or both, while tracking performance is consistently improving. The control and adaptation laws associated with SRCSys are provided in (Slotine and Li, 1991; Maghareh et al., 2020).

### State Estimation, Model Updating, and Uncertainty Quantification

To facilitate run-time model updating and uncertainty quantification, and to eventually develop run-time performance indicators, strategies to improve computational efficiency are essential. RTHS experiments are frequently performed to study unexpected behavior or a part of the physical substructure may experience a failure, drastically changing its dynamics. Such a change will impact the safety and integrity of the experiment. Therefore, the run-time model updating and uncertainty quantification methods adopted must be able to handle such large variations in behavior. The remainder of this section presents the choices of the particle filter for model updating and uncertainty quantification in REFORM-I.

Various strategies are available for run-time model updating and uncertainty quantification. One conventional approach is to apply an optimization strategy to identify the parameters that minimize the difference between the predicted and measured model properties (e.g., residual-based model updating by Li et al., 2018). However, most of these model updating methods are deterministic, and do not consider uncertainties in the measurements or in the model adopted. In contrast, methods grounded in Bayesian inference determine the distribution of each of the uncertain model parameters based on observations (Zarate et al., 2012). Thus, they not only offer the ability to estimate the model parameters, but they directly facilitate rigorous uncertainty quantification for those parameters and/or states. However, due to high computational cost of such methods, and the lack of efficient sampling schemes using Monte Carlo simulation, they are quite limited for use during run-time in most RTHS experiments (Yang and Lam, 2018). Alternatively, various Bayesian filtering techniques have the potential to support both model updating and uncertainty quantification at run-time (Sarkka, 2013). Typical examples of such techniques include the extended Kalman filter, unscented Kalman filter, and particle filter. After initialization, this class of algorithms support predictive capabilities to estimate the distribution of model parameters or responses at each time step, and update the posterior distribution based on observed data.

**FIGURE 5 |:**
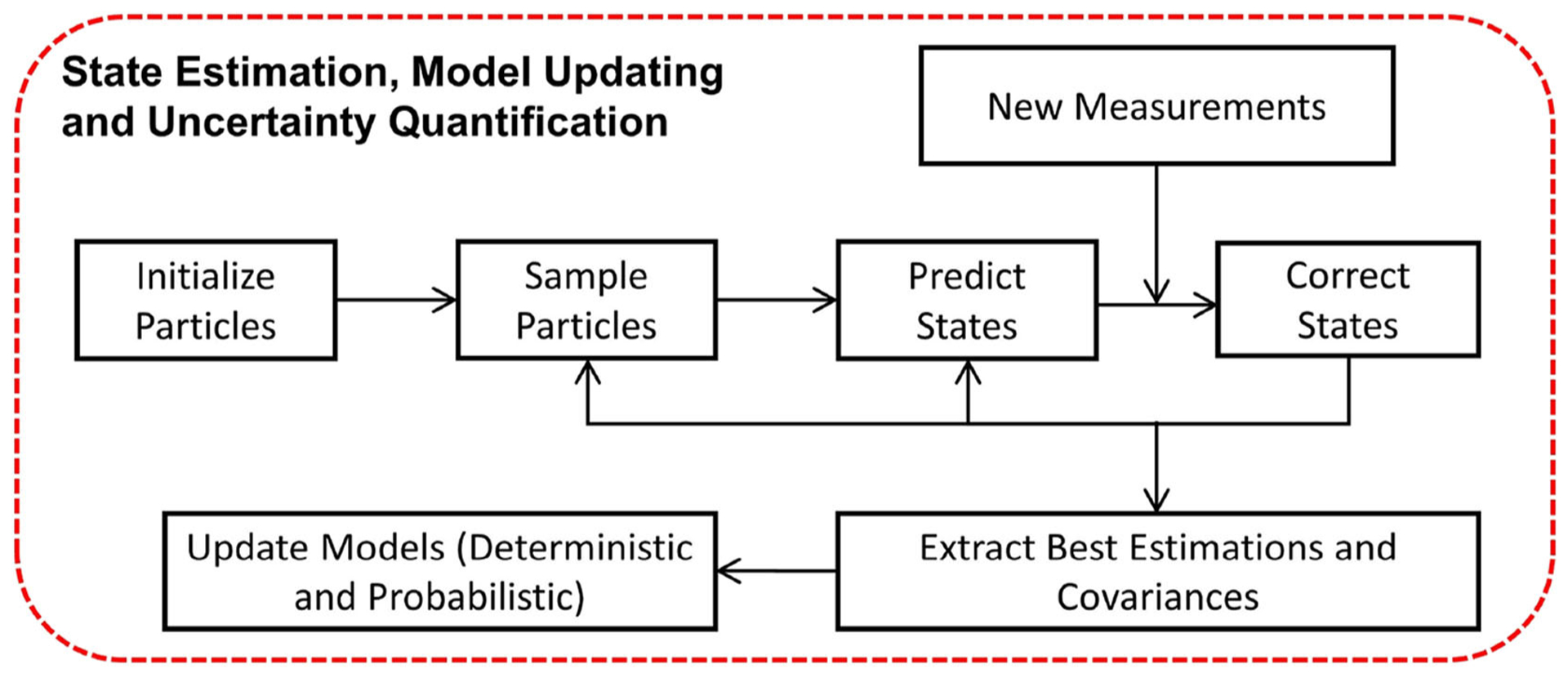
Workflow of particle filter for model updating and uncertainty quantification.

The particle filter is a non-parametric, recursive Bayesian state estimator. It represents the posterior probability density function of the states as samples, or particles, with associated weights (Aru, 2007). In this building block, the particle filter is used for parameter estimation, uncertainty quantification, and response prediction, facilitating run-time model updating. The workflow of the particle filter block is illustrated in Figure 5. The particle filter is first initialized by assigning the number of particles and initial conditions (state and covariance). The particles are then sampled from a Gaussian distribution representing the prior density function. Afterwards, particles predict the next state based on an appropriate state transition function. For an illustrative example of the use of the particle filter for parameter identification in highly non-linear systems (see Condori-Uribe et al., 2019).

To enable black-box RTHS and accommodate extensive variations in the physical specimen, such as non-stationary behavior or component failure, non-linear estimators (e.g., unscented Kalman filters, particle filters) are needed. Run-time execution of such filters enables: (1) estimation of the states and updating of the parameters of the physical specimen model for SRCSys implementation; and (2) monitoring, in real time, of the overall performance of the dynamic simulation. In REFORM-I, real-time non-linear estimators are developed by adopting a particle filter algorithm that takes advantage of measured displacement and force signals. The particle filter algorithm developed for this building block can serve multiple purposes including: parameter estimation (a.k.a. model updating), uncertainty quantification, and state estimation.

This approach is shown to significantly improve the performance of the real-time control system and accommodates extensive variations in the behavior of the physical specimen (Condori-Uribe et al., 2019). In addition, it enables run-time estimation and associated uncertainties of the model parameters, thus serving as the basis for *run-time sensitivity indicators* and *run-time sensitivity envelope*. However, including computationally-intensive estimation techniques does necessitate the use of the multi-rate coordination block for run-time execution. Further details on the state estimation building block for non-linear systems using the particle filter are provided in Condori-Uribe et al. (2019).

### Run-Time Sensitivity Indicator and Stability Threshold

An essential building block in REFORM-I is the run-time thresholds and indicators to facilitate safe and accurate experiments. Stability and accuracy of a particular RTHS configuration are mainly functions of four factor: (1) overall dynamics of the reference structure; (2) fidelity of the computational substructure (related to computational workload and simulation rate); (3) partitioning configuration; and (4) capability to implement interface conditions (related to control workload and simulation rate) (Maghareh et al., 2017).

In this building block, we have established run-time sensitivity indicator, run-time sensitivity envelope, and run-time stability threshold based on the first, third, and fourth factors. To compute the sensitivity indicator, envelope, and the stability threshold on-the-fly, we (1) use updated parameters and associated uncertainties of the physical substructure (from the non-linear estimator in the preceding section); (2) evaluate tracking performance at the interface; and (3) compute critical time delay.

**FIGURE 6 |:**
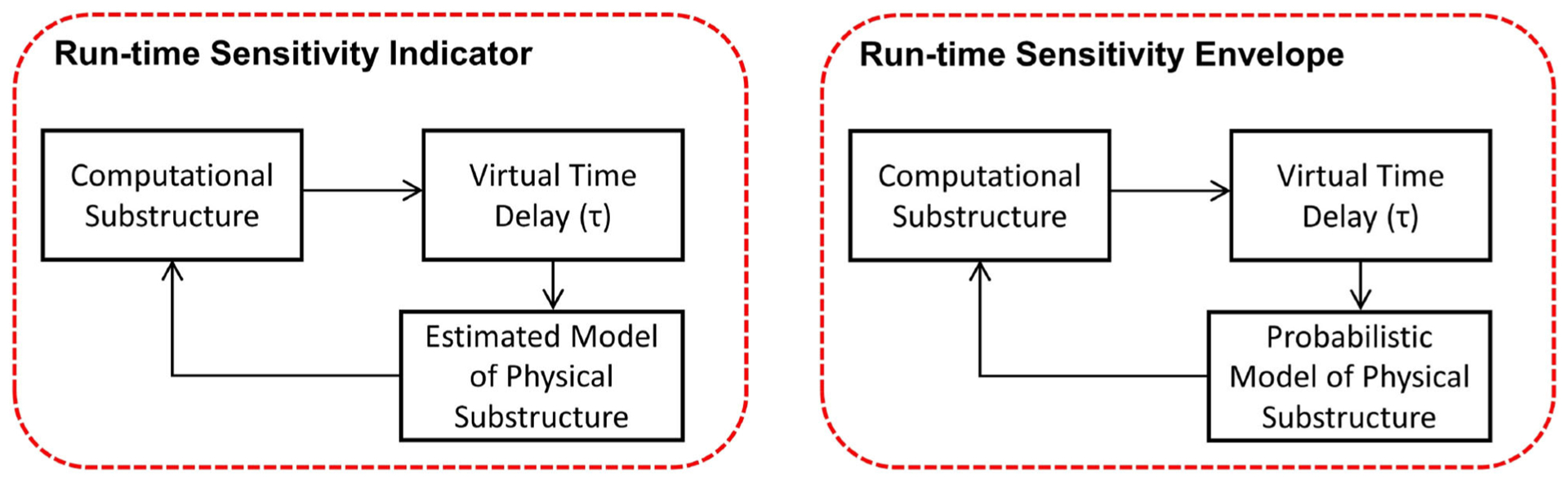
Run-time sensitivity analysis: frameworks associated with run-time sensitivity indicator and run-time sensitivity envelope.

**FIGURE 7 |:**
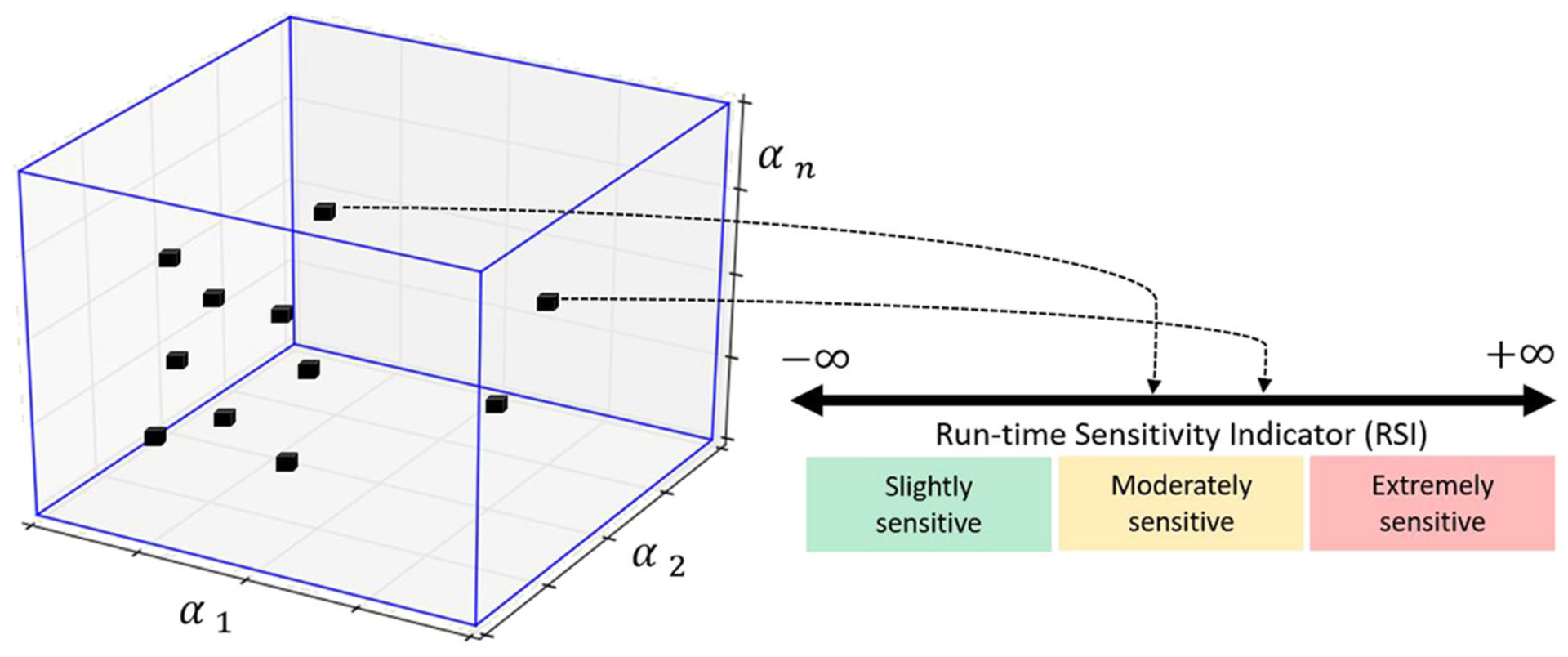
Geometric representation of mapping from an n-dimensional hyperplane associated with varying parameters of the physical substructure model to the RSI. Prior to conducting an experiment, the range of possible values of the RSI is classified, on the basis of system instability as extremely sensitive (red), moderately sensitive (yellow), and slightly sensitive (green) (Maghareh et al., 2017; Silva et al., 2020).

In REFORM-I, we developed the run-time sensitivity indicator based on the knowledge gained in developing predictive (before any testing) stability and performance indicators intended to support the design of challenging RTHS experiments (Maghareh et al., 2014, 2017). For these predictive indicators, the sensitivity in partitioning of any linear or piecewise linear partitioned system to interface desynchronization is computed before the testing. The run-time sensitivity indicator (and run-time sensitivity envelope), however, are computed based on the sensitivity of the potential partitioning associated with the computational substructure and the estimated model (and associated uncertainties) of the physical substructure in real-time. To compute these in real-time, we: (1) characterize the potential critical time delay (i.e., the delay associated with the first occurrence of stability switch in Figure 6) associated with a wide and specific range for parametric variations (*α*_1_, *α*_2_,… *α*_n_, see Figure 7) in the potential physical substructure model using the method developed in Maghareh et al. (2017); (2) update the physical substructure model in real-time using a particle filter; (3) update the uncertainties associated with these parameters; (4) compute, on the fly, the critical time delays (*τ*_*cr*_) associated with virtual frameworks shown in Figure 6; and, (5) compute the run-time sensitivity indicator (RSI) and run-time envelope as follows:

(4) $$\text{RSI}=-{\text{log}}_{10}\left[\tau_{\text{cr}}(\text{msec})\right]$$

Equation (4) maps *τ*_*cr*_ *ϵ* (0, ∞) to the RSI *ϵ* (−∞, ∞). To calculate the run-time sensitivity envelope, however, a vector of run-time sensitivity indicators (associated with the updated range of parametric variations in Figure 6 obtained from the particle filter) are computed using Equation (4) and the envelope is constructed based on the maximum and minimum values at each time step.

The run-time stability threshold, however, evaluates tracking performance, in real-time, at the interface between the physical and computational substructures. This threshold is based on two factors: (1) the control strategy, and (2) the actual and instant sensitivity of the experiment. The latter is estimated using the RSI. The remainder of this section discusses the adopted strategy to monitor the run-time stability of the experiment using SRCSys.

In SRCSys, we have broken down the tasks associated with the non-linear tracking problem into two parts (Slotine and Li, 1991; Maghareh et al., 2020): (1) the control law is designed based on defining a compact tracking error. The compact tracking error creates a time-varying hyperplane called boundary layer. As a part of developing the control law, the time-varying boundary layer is designed to be an invariant set; (2) once the time-varying boundary layer is reached, the tracking error is determined, bounded and stability is guaranteed based on Barbalat’s lemma (Slotine and Li, 1991).

**FIGURE 8 |:**
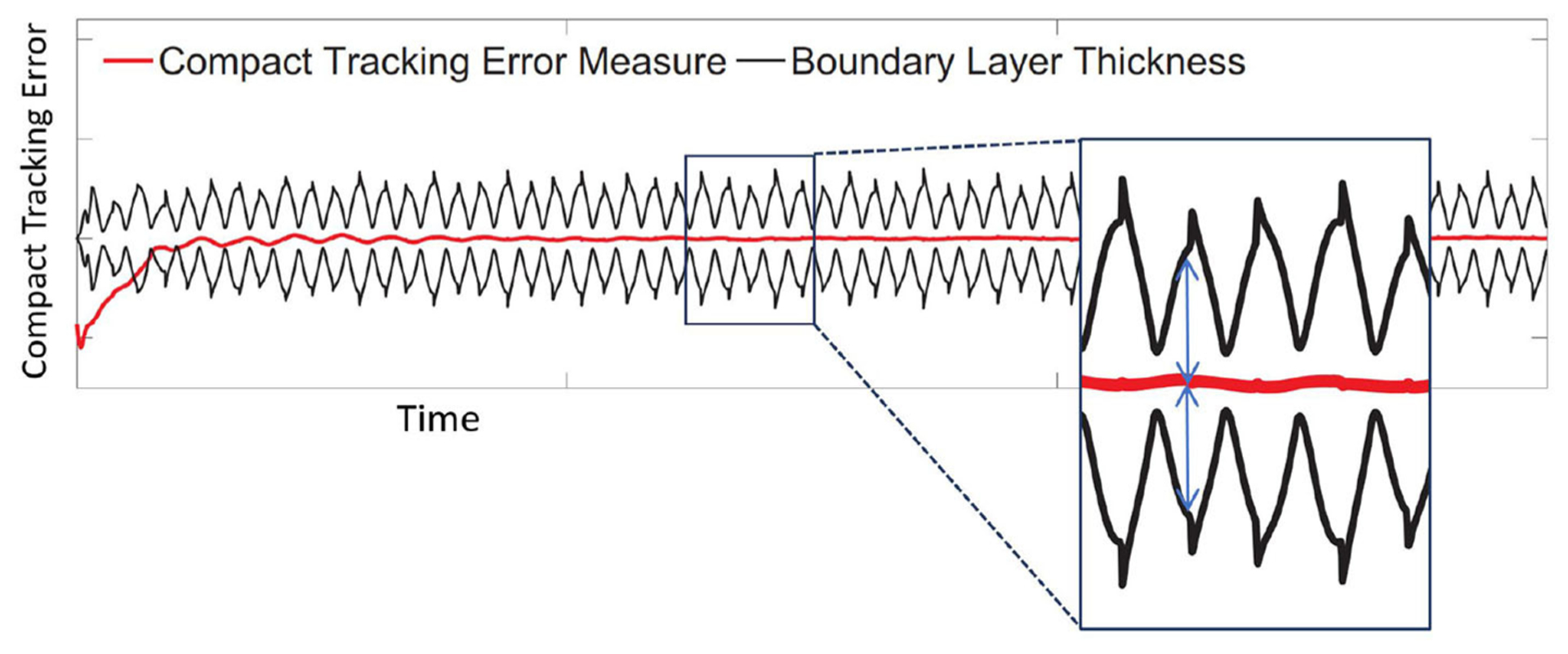
Real-time computation and visualization of the run-time stability threshold.

The control law in the SCRSys is based on the assumption that the uncertainties associated with the dynamics of the plant are bounded. This assumption is aligned with our goal of determining the specific ranges of parametric variations in developing the RSI. In other words, the design of the SCRSys control law also specifies the ranges of parametric variations associated with the physical specimen. During an RTHS experiment, the boundary layer thickness and the compact tracking error over time convey a significant amount of information about whether the specific ranges of parametric variations are suitably chosen or not. A violation of this threshold, i.e., when the compact tracking error breaches the boundary layer, indicates that the stability of the control loop (and therefore the entire simulation) may be compromised. Thus, in REFORM-I the run-time stability threshold is a time-varying metric which is monitored in real-time and computed as the minimum distance between the compact tracking error and the boundary layer. Figure 8 demonstrates the real-time computation and visualization of the run-time stability threshold.

REFORM-I is designed to provide a robust control framework without compromising the stability and safety of an RTHS experiment. Together, the run-time stability threshold, the run-time sensitivity indicator and envelope serve as the foundation for evaluating the integrity and safety of an RTHS experiment. During an RTHS experiment using REFORM-I, should the run-time stability threshold become negative, the emergency stop function would be activated and will decouple the computational and physical substructures through switching off the ground excitation signal and the feedback force from the physical substructure to the computational substructure. Thus, the entire simulation will become a simple tracking control problem which tracks a smooth decay signal which is associated with the unforced response of the computational substructure. The purpose of the emergency stop function is to ensure a stable experiment, suppressing any situation in which large actuators could potentially generate dangerous physical instabilities in the lab environment. During an experiment, run-time indicators are used to predict such behavior and trigger the emergency stop function. Upon the activation of the emergency stop, the computational substructure will be decoupled from the transfer system. At the same time, the command signal(s) to the hydraulic actuator(s) will follow a smooth decay signal(s) to a safe state.

## NUMERICAL VALIDATION

The RTHS benchmark control problem (Silva et al., 2020) is employed for illustration and numerical validation of the building blocks that comprise REFORM-I. This section is structured as follows. First, we provide a brief overview of the benchmark problem. Next, we describe the three numerical studies considered in this section, and their corresponding virtual-RTHS (vRTHS, hereafter) results.

### The Benchmark Control Problem for RTHS

**FIGURE 9 |:**
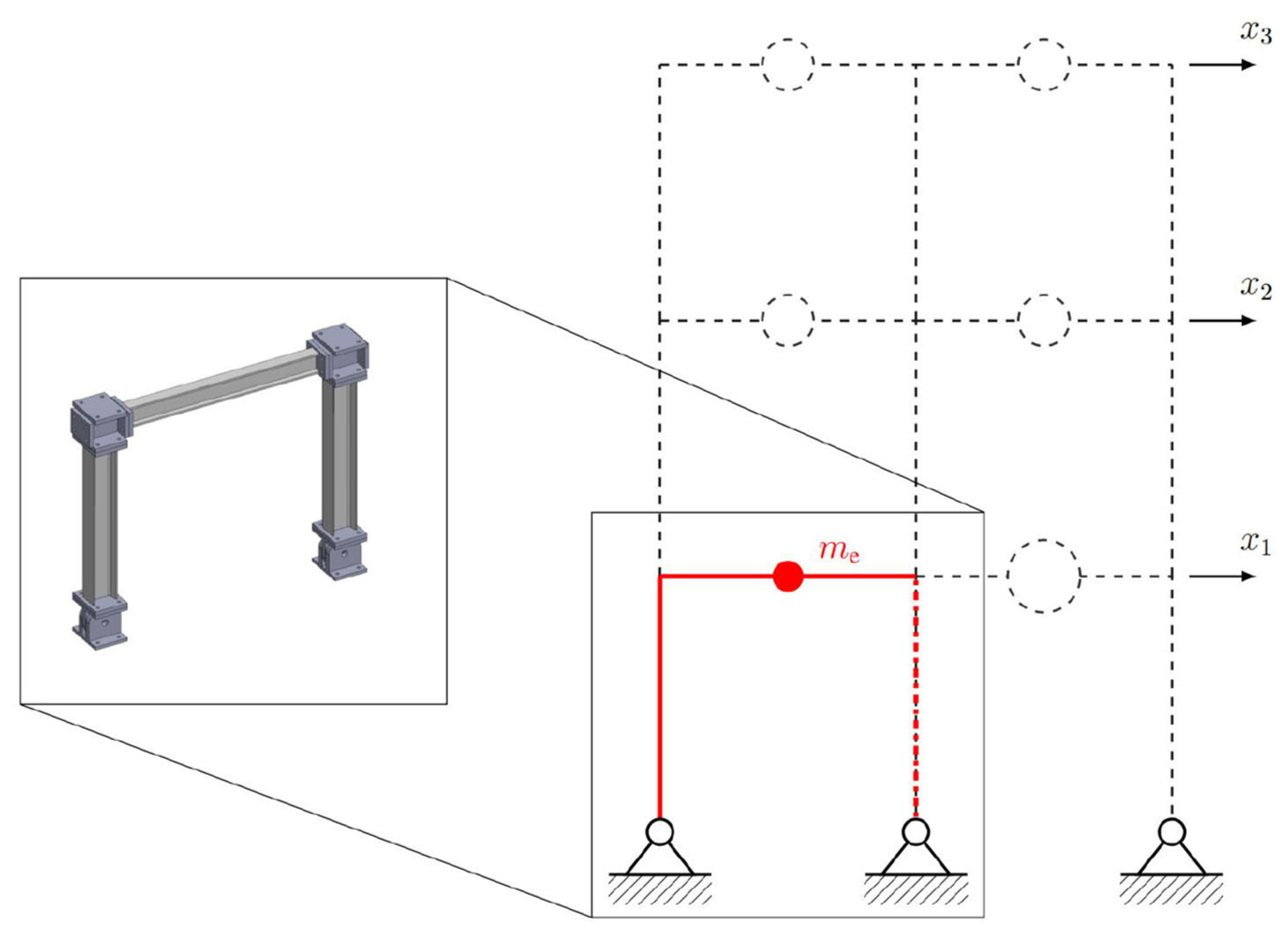
Numerical and experimental substructures of benchmark structure by Silva et al. (2020).

The benchmark control problem considers a laboratory model of a typical frame structure. The model has three stories and two bays, has pinned connections at the base, and moment-resisting connections with strong-column weak-beam design. A two-dimensional finite element (FE) model is developed using linear elastic models. Simplifying assumptions are made to reduce the order of the FE model from 30 DOF to 3 DOF, lowering the computational complexity of the model. The equation of motion for the reduced order model is referred to as the reference model,

(5) $${\text{M}}_{\text{r}}\ddot{\text{x}}+{\text{C}}_{\text{r}}\dot{\text{x}}+{\text{K}}_{\text{r}}\text{x}=-{\text{M}}_{\text{r}}\iota {\ddot{\text{x}}}_{\text{g}}$$

where M_r_, C_r_, and K_r_ are mass, stiffness, and damping matrices of the reference model, respectively. ẍ_*g*_ denotes ground acceleration and x, ẋ and ẍ are displacement, velocity, and acceleration vectors, respectively. The reference model is then partitioned into numerical and experimental substructures as shown in Figure 9. The red solid lines represent the experimental substructure. Accordingly, the partitioned equation of motion can be expressed as

(6) $${\text{M}}_{\text{n}}\ddot{\text{x}}+{\text{C}}_{\text{n}}\dot{\text{x}}+{\text{K}}_{\text{n}}\text{x}=-{\text{M}}_{\text{r}}\iota {\ddot{\text{x}}}_{\text{g}}-\underset{\text{fe}}{\underset{︸}{({\text{M}}_{\text{e}}\ddot{\text{x}}+{\text{C}}_{\text{e}}\dot{\text{x}}+{\text{K}}_{\text{e}}\text{x}}}).$$

where the (·)n and (·)e subscripts refer to the computational and physical substructures, respectively. System identification is performed using the experimental data to obtain the experimental structural mass (*m*_*e*_ = 29:1 kg), lateral stiffness (*k*_*e*_ = 1:19 × 10^6^ N/m), and damping coefficient (*c*_*e*_ = 114:6 N:s/m). Since the experimental substructure is fixed, several partitioning cases are defined by varying the structural parameters of the reference structure, as shown in Table 1. Variations are considered in both the modal damping and the mass of each floor of the reference structure, thus yielding different stability and performance scenarios. Using the predictive stability indicator and the proposed sensitivity classifications (Maghareh et al., 2017), the partitioning configurations associated with Cases 1–3 fall within the slightly sensitive class, while Case 4 falls within the moderately sensitive class. Thus, Cases 1–3 are less sensitive to desynchronization at the interface as compared to Case 4. Further, the predictive indicators reveal that when using the same transfer system control strategy, a researcher should expect somewhat less accuracy in the results associated with Case 4 compared to the results from Cases 1–3. It should be noted that this paper only studies the extreme ones, Case 1 (i.e., the least sensitive case to desynchronization) and Case 4 (i.e., the most sensitive case to desynchronization).

**FIGURE 10 |:**
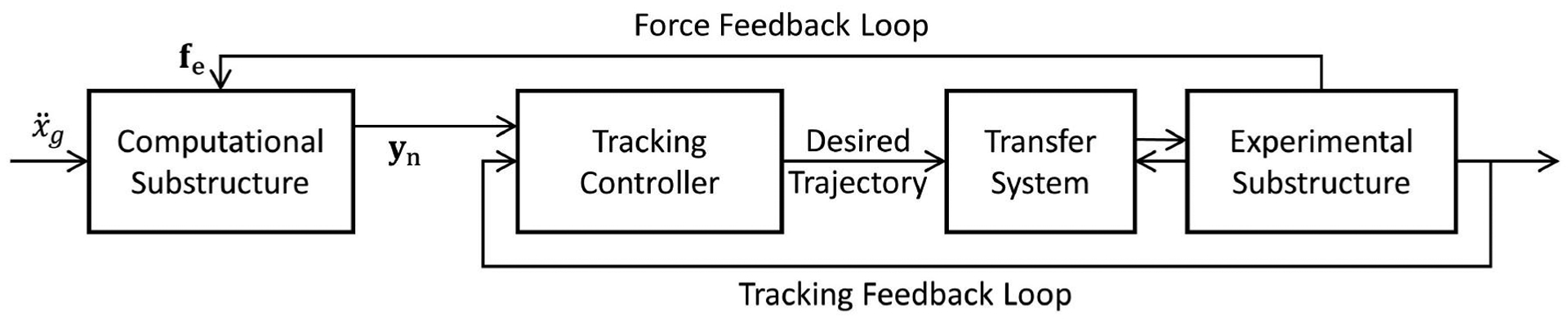
RTHS implementation for benchmark problem by Silva et al. (2020).

Figure 10 shows the implementation of RTHS using a sample control strategy [i.e., a phase-lead compensator and a proportional-integral (PI) controller] provided by Silva et al. (2020) to demonstrate the problem. The ground input (ẍg) and the feedback force vector (f_e_) are the inputs of the numerical substructure whose 296 output (y_n_) is fed into the tracking controller. The controller generates a command signal to the transfer system. The force at the first floor is measured and fed back to the numerical substructure to construct the force feedback loop (see Equation 6). The displacement output of the experimental substructure is used to form the tracking feedback loop.

### Virtual RTHS Studies and Results

This section presents the numerical validation of the building blocks provided in REFORM-I. Because this phase is focused on developing a *modular framework* to enable conducting experiments *safely and with high confidence* by dedicating the appropriate resources to perform the control and prediction tasks, we perform three vRTHS studies using REFORM-I. Study 1 focuses on Cases 1 and 4 in the benchmark problem, as defined previously and in the original problem statement. Then in Studies 2 and 3 we consider a sudden component failure during a vRTHS of Cases 1 and 4. In Study 2 and Study 3, we artificially define a story drift threshold (4.4 mm) at which a failure of one of the experimental columns in the benchmark structure occurs (i.e., *k*_*e*_ drops by 50%). In Studies 1 and 2, we implement the sample linear control strategy provided in the benchmark problem. However, in Study 3, the building blocks provided in REFORM-I are activated to enhance the safety and integrity of the simulation. To quantitatively evaluate the performance of each study, we utilize a cumulative normalized error indicator which is a global simulation performance indicator. The cumulative normalized error is defined as follows:

(7) $${\text{CNE}}_{1}(\text{w})=\frac{1}{\text{max}\left(\left|x_{1}^{\text{ref}}\right|\right)}\sum_{i=1}^{w}\left(\left|x_{1}^{\text{vRTHS}}(i)-{\text{x}}_{1}^{\text{ref}}(i)\right|\times \Delta t\right)$$

where CNE_1_, $x_{1}^{\text{vRTHS}}$, ${\text{x}}_{1}^{\text{ref}}$, and Δ*t* refer to cumulative normalized error associated with the first floor, virtual-RTHS displacement at the first floor, reference displacement at the first floor, and sub-interval time step which is associated with displacement measurement. Note the cumulative normalized error is extremely sensitive to time desynchronization between the two signals which makes it a suitable error indicator for stability analysis of different systems and configurations.

**FIGURE 11 |:**
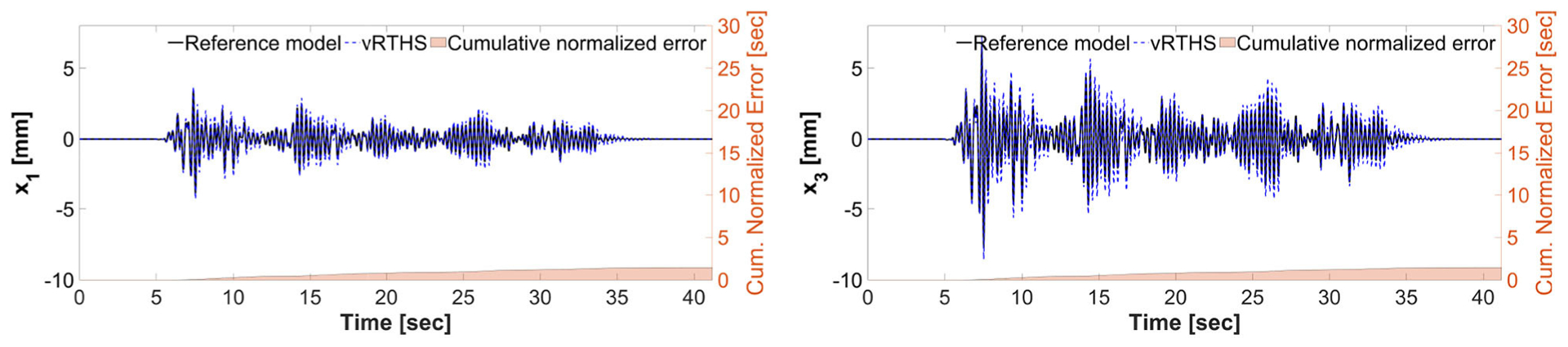
Study 1 (Case 1): vRTHS response comparisons of the 1st and 3rd floor displacements under El Centro excitation.

**FIGURE 12 |:**
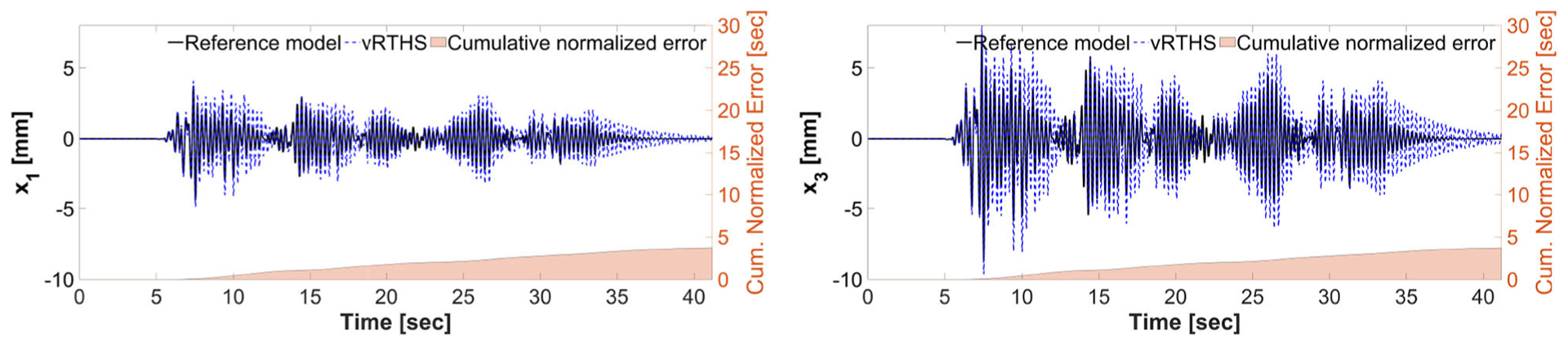
Study 1 (Case 4): vRTHS response comparisons of the 1st and 3rd floor displacements under El Centro excitation.

Figures 11, 12 show the reference model responses, the vRTHS responses and cumulative normalized errors associated with Study 1 (Case 1) and Study 1 (Case 4), respectively. These results match those provided in the RTHS benchmark control problem by Silva et al. (2020). As noted earlier, the results show that the predictive stability indicator (PSI) associated with Case 1 (PSI = 1.17) is greater than that associated with Case 4 (PSI = 0.94), confirming that using the same transfer system control strategy leads to greater global accuracy in Case 1 as compared to Cases 4.

**FIGURE 13 |:**
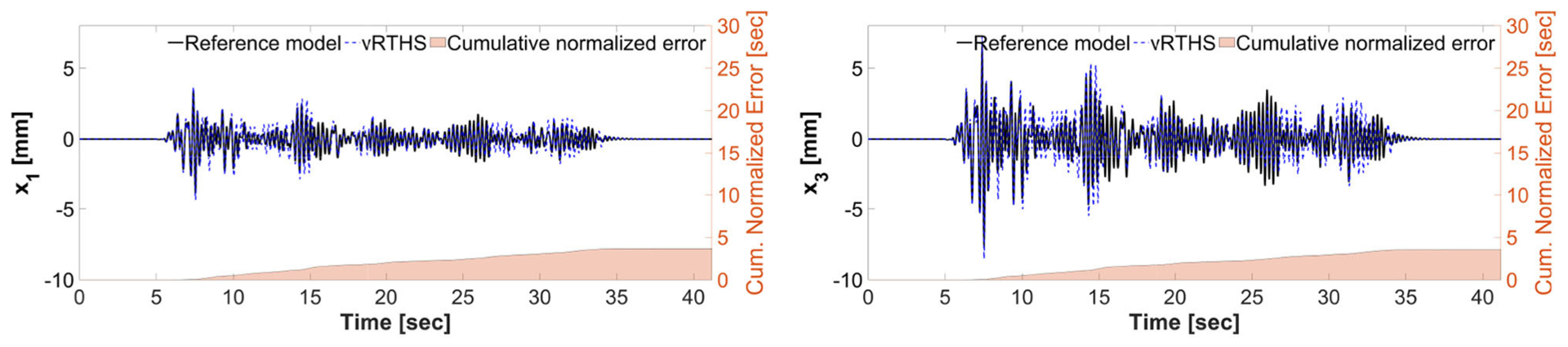
Study 2 (Case 1): virtual-RTHS response comparisons of the 1st and 3rd floor displacements under El Centro excitation.

**FIGURE 14 |:**
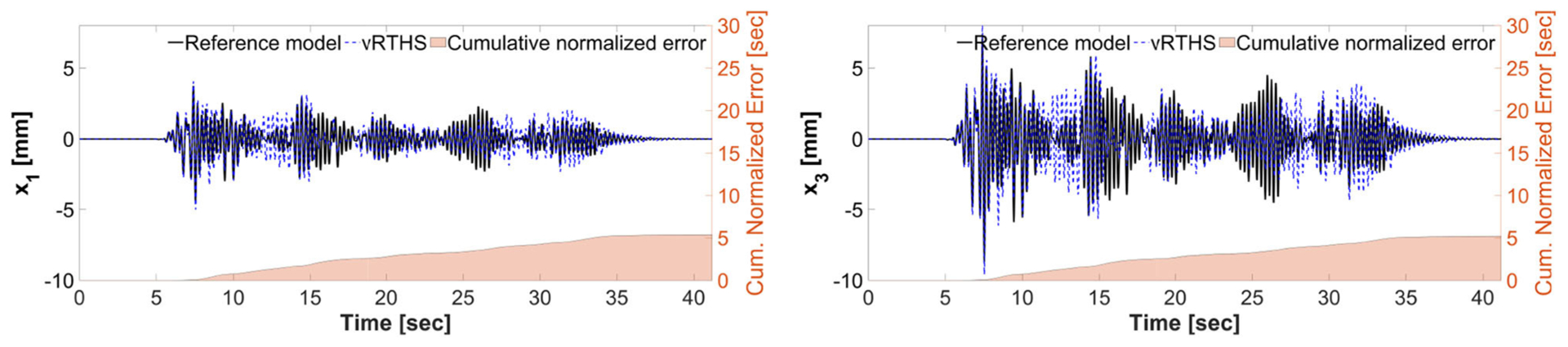
Study 2 (Case 4): vRTHS response comparisons of the 1st and 3rd floor displacements under El Centro excitation.

Figures 13, 14 show the reference model responses, the vRTHS responses and cumulative normalized errors associated with Study 2 (Case 1) and Study 2 (Case 4), respectively. In these simulations the lateral stiffness associated with the experimental substructure drops by 50% at 7.53 and 7.48 s, respectively. The results confirm that Case 1 [PSI (before damage) = 1.17, PSI (after damage) = 1.50] is less sensitive to desynchronization at the interface as compared to Case 4 [PSI (before damage) = 0.94, PSI (after damage) = 1.25]. In addition, a comparison of the results between Study 1 and Study 2 shows that although the damage creates less sensitive configurations [compare PSI (before damage) and PSI (after damage)], the global performance of Study 1 is slightly more accurate than that of Study 2. This observation can be attributed to the fact that the sample linear controller provided in the RTHS benchmark problem definition lacks the level of robustness and adaptation required to accommodate extensive variations during a real-time hybrid simulation.

**FIGURE 15 |:**
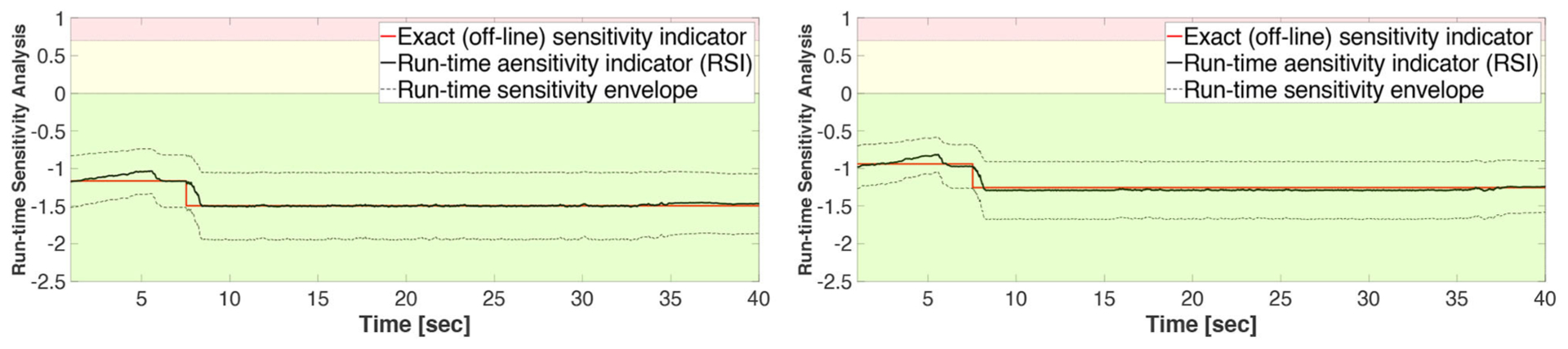
Study 3 (Case 1 and Case 4): exact (off-line) indicators, run-time sensitivity indicators, and corresponding envelopes.

**FIGURE 16 |:**
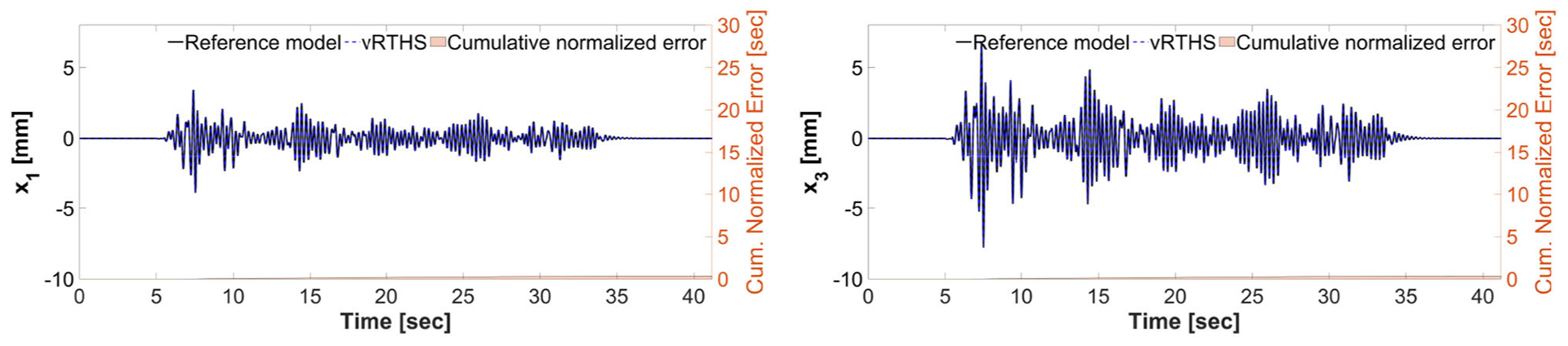
Study 3 (Case 1): vRTHS response comparisons of the 1st and 3rd floor displacements under El Centro excitation.

**FIGURE 17 |:**
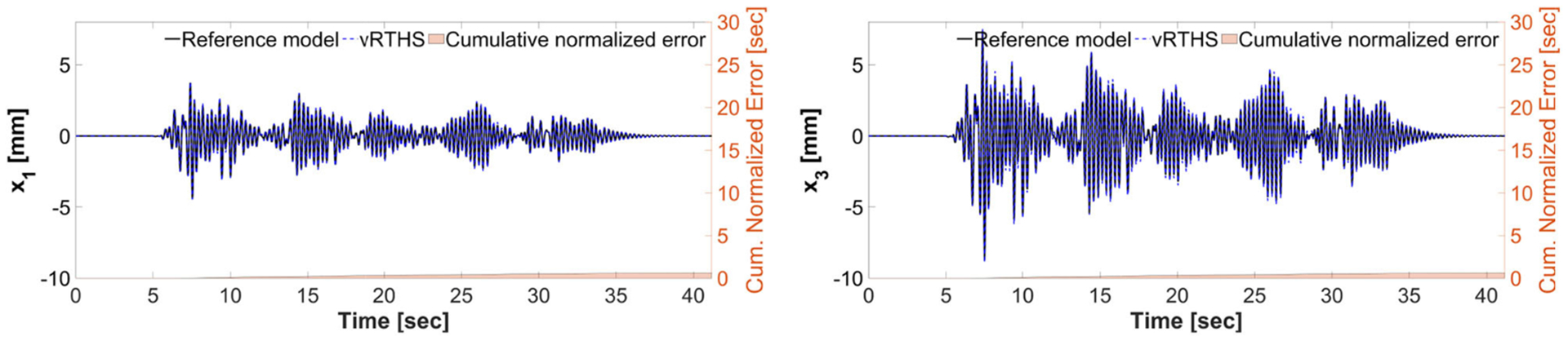
Study 3 (Case 4): vRTHS response comparisons of the 1st and 3rd floor displacements under El Centro excitation.

Figures 15–17 show the results of Study 3 (Case 1 and Case 4). In these simulations the lateral stiffness associated with the experimental substructures drops by 50% at 7.61 and 7.72 s, respectively. Figure 15 shows the exact (off-line) sensitivity indicators, run-time sensitivity indicators, and run-time sensitivity envelopes associated with Cases 1 and 4. Prior to conducting the simulations, the range of possible values of the RSI is classified, on the basis of system instability as extremely sensitive (red, RSI > 0:7), moderately sensitive (yellow, 0 <= RSI <= 0:7), and slightly sensitive (green, RSI < 0), see Figures 6, 15.

These results also demonstrate that Case 1 is less sensitive to desynchronization at the interface both before and after the damage event. The exact sensitivity indicators are computed off-line using (1) the exact time of damage occurrence, and (2) full knowledge of the physical substructure parameters both before and after the damage occurrence. However, in an RTHS experiment neither will be available before the test begins. Therefore, we use (a) the model updating building block to estimate, on-the-fly, the parameters of the physical substructure and (2) compute and monitor, in real-time, the sensitivities of the simulations before and after the occurrence of the damage. Aligned with our observation in Study 2, Figure 17 also confirms that the failure of the column makes the simulation less sensitive to desynchronization at the interface. However, unlike the poor global performance results associated with Study 2, the results of Study 3 provided in Figures 16, 17 show significant improvements due to the built-in adaptation and robustness capabilities within REFORM-I.

Figure 15 shows that the exact sensitivity indicator falls within the run-time sensitivity envelope. This outcome is a critical requirement in case any extensive variations occur within the physical substructure (e.g., component failure, bifurcation, non-stationary dynamics, and dynamical switching), leading to more sensitivity to desynchronization at the interface. Whether or not the envelope can capture the exact sensitivity of the simulation will depend on the robustness of the run-time model updating and estimation building block.

## CONCLUSIONS

Despite the potential for using RTHS to conduct cost-effective experiments at scale, the hazards engineering community has not been able to fully exploit this realistic testing method. The lack of systematic procedures and stringent requirements for the safety, integrity, and coordinated evolution of an RTHS test (during the experiment) and measures for the accuracy of experimental results (both during and after the experiment) have prevented researchers from tackling many problems that are of great interest to the hazards community. For instance, RTHS of systems with component damage or failure require advanced methods that can assess the system and adapt to changing parameters, necessitating run-time estimation of the new parameters and the associated uncertainty. In response to this technical hurdle, REFORM is developed to provide a modular framework that will enable conducting more challenging and realistic experiments safely and with high confidence. REFORM-I (this first phase of REFORM development and numerical validation) serves as a foundation for extending RTHS application to black-box RTHS experiments: a general physical substructure with unanticipated dynamical behaviors. Herein we describe and demonstrate the building blocks and the numerical validation considered within REFORM-I. These building blocks (i.e., run-time sensitivity indicator, run-time stability threshold, run-time state-estimation and model updating technique, multi-rate coordination, and self-tuning robust control system) which are grounded in non-linear control and estimation theories, provide both mechanisms to support adaptation and robustness and the knowledge to use them effectively for conducting RTHS experiments. The methods are demonstrated and validated using a well-known benchmark problem in RTHS.

## Figures and Tables

**TABLE 1 |:** Virtual-RTHS partitioning cases proposed by Silva et al. (2020).

Partitioning configuration	Reference floor mass (kg)	Reference modal damping (%)
Case 1	1,000	5
Case 2	1,100	4
Case 3	1,300	3
Case 4	1,000	3
